# Retroperitoneoscopic pyelolithotomy: a good alternative treatment for renal pelvic calculi in children

**DOI:** 10.1590/S1677-5538.IBJU.2015.0242

**Published:** 2016

**Authors:** Bruno Nicolino Cezarino, Rubens Park, Paulo Renato Marcelo Moscardi, Roberto Iglesias Lopes, Francisco T. Denes, Miguel Srougi

**Affiliations:** 1 Departamento de Urologia, Faculdade de Medicina da Universidade de São Paulo, SP, Brasil

## Abstract

**Introduction:**

Nephrolitiasis, once considered an adult disease, has become increasingly prevalent in children, with an increase from 6% to 10 % annually in past 25 years. Kidney stones in pediatric population can result from metabolic diseases in up to 50% of children affected. Other factors associated with litiasis are infection, dietary factors, and anatomic malformations of urinary tract.

Standard treatment procedures for pediatric population are similar to adult population. Extracorporeal shock wave lithotripsy (ESWL), ureterorenoscopy (URS), percutaneous nepfrolithotomy (PCNL), as well as laparoscopic and retroperitoneoscopic approaches can be indicated in selected cases.

The advantages of laparoscopic or retroperitoneoscopic approaches are shorter mean operation time, no trauma of renal parenchyma, lower bleeding risk, and higher stone-free rates, especially in pelvic calculi with extrarenal pelvis, where the stone is removed intact.

**Patient and Methods:**

A 10 year-old girl presented with right abdominal flank pain, macroscopic hematuria, with previous history of urinary infections‥ Further investigation showed an 1,5 centimeter calculi in right kidney pelvis. A previous ureterorenoscopy was tried with no success, and a double J catheter was placed.

After discussing options, a retroperitoneoscopic pielolithotomy was performed.

**Results:**

The procedure occurred with no complications, and the calculi was completely removed. The foley catheter was removed in first postoperative day and she was discharged 2 days after surgery. Double J stent was removed after 2 weeks. Conclusions: Retroperitoneoscopic pielolithotomy is a feasible and safe procedure in children, with same outcomes of the procedure for adult population.

## ARTICLE INFO

**Available at:** **www.intbrazjurol.com.br/video-section/cezarino_1248_1248**

**Int Braz J Urol. 2016; 42 (Video #11): 1248-48**

